# Socio Economic Status and Traumatic Brain Injury amongst Pediatric Populations: A Spatial Analysis in Greater Vancouver

**DOI:** 10.3390/ijerph121215009

**Published:** 2015-12-08

**Authors:** Ofer Amram, Nadine Schuurman, Ian Pike, Natalie L Yanchar, Michael Friger, Paul B. McBeth, Donald Griesdale

**Affiliations:** 1 Department of Geography, Simon Fraser University, 8888 University Drive, Burnaby, BC V5A 1S6, Canada; nadine@sfu.ca; 2 Department of Pediatrics, Faculty of Medicine, University of British Columbia, Vancouver, BC V6T 1Z4, Canada; BC Injury Research and Prevention Unit, Child and Family Research Institute, BC Children’s Hospital, Vancouver V6H 3V4, Canada; ipike@cw.bc.ca; 3 Department of Surgery, Dalhousie University, Halifax, NS B3H 4R2, Canada; Natalie.Yanchar@iwk.nshealth.ca; 4 Faculty of Health Science, Ben Gurion University, Beer Sheva 8410501, Israel; friger@bgu.ac.il; 5 Departments of Surgery and Critical Care Medicine, Alberta Health Services, Calgary, AB T6G 2R3, Canada; pmcbeth@gmail.com; 6 Department of Anesthesiology, Pharmacology & Therapeutics, University of British Columbia, Vancouver, BC V6T 1Z4, Canada; Donald.Griesdale@vch.ca

**Keywords:** traumatic brain injury, pediatric injury, injury hotspot, injury prevention, geographic information systems

## Abstract

*Introduction*: Within Canada, injuries are the leading cause of death amongst children fourteen years of age and younger, and also one of the leading causes of morbidity. Low Socio Economic Status (SES) seems to be a strong indicator of a higher prevalence of injuries. This study aims to identify hotspots for pediatric Traumatic Brain Injury (TBI) and examines the relationship between SES and pediatric TBI rates in greater Vancouver, British Columbia (BC), Canada. *Methods*: Pediatric TBI data from the BC Trauma Registry (BCTR) was used to identify all pediatric TBI patients admitted to BC hospitals between the years 2000 and 2013. Spatial analysis was used to identify hotspots for pediatric TBI. Multivariate analysis was used to distinguish census variables that were correlated with rates of injury. *Results*: Six hundred and fifty three severe pediatric TBI injuries occurred within the BC Lower Mainland between 2000 and 2013. High rates of injury were concentrated in the East, while low rate clusters were most common in the West of the region (more affluent neighborhoods). A low level of education was the main predictor of a high rate of injury (OR = 1.13, 95% CI = 1.03–1.23, *p*-Value 0.009). *Conclusion*: While there was a clear relationship between different SES indicators and pediatric TBI rates in greater Vancouver, income-based SES indicators did not serve as good predictors within this region.

## 1. Introduction

Within Canada, injuries are the leading cause of death amongst children fourteen years of age and younger and also one of the leading causes of morbidity. Over the last five decades, Canada, like many other western countries, has seen a 50% decline in childhood injuries [1]; however, low socio economic status (SES) seems to be a strong indicator of a higher prevalence of injuries among children [2,3,4]. Despite a declining absolute number of injuries across the SES spectrum in recent years, emerging research shows a widening in the gap between rich and poor in terms of injury prevalence [1]. 

Injuries are unique in that they are always externally caused. As a result, they are directly influenced by the physical and social environment [5]. Physical and social environments can influence the injury rates in a variety of ways. For example, children of low SES and students attending schools in low SES neighbourhoods experience a greater number of severe injuries [6]. Children with low SES typically live in older, less well-maintained homes and may, as a consequence, face a greater risk of exposure to lead poisoning and house fires [7]. Epidemiological studies examining the relationship between SES and injury rates typically use various measures of SES. The most common are income, employment, level of education, housing, and demographics. SES variables can also be combined by creating a deprivation index [8]. 

Traumatic Brain Injuries (TBI) are the most common cause of mortality amongst pediatric patients, causing approximately 75% of the injury-related deaths [9]. Patients recovering from TBI are also likely to have some type of long term disability. In fact, approximately half of these patients will develop cognitive, emotional or behavioural problems [10]. In Canada, the financial cost of recovery from TBI is estimated at $93,000 in the first year alone [11]. The high cost of recovery, coupled with the higher incidence of TBI amongst children and families with low socio economic status (SES), suggests that TBI may have a greater impact on this population. [12,13].

The study reported here identified hotspots for pediatric TBI and examined the relationship between SES and pediatric TBI rates in greater Vancouver, British Columbia (BC), Canada. More specifically, the study sought to identify those census variables most closely associated with high rates of pediatric TBI.

## 2. Methods

### 2.1. Data

**Figure 1:**
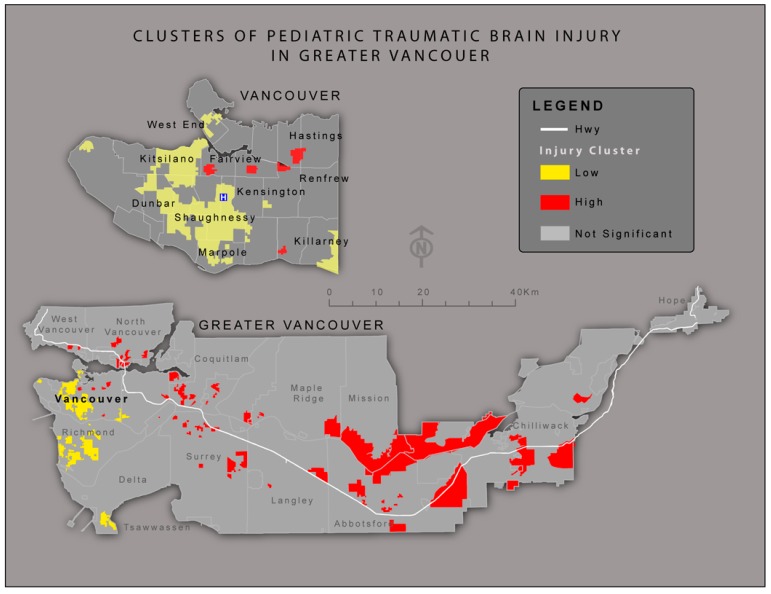
Map showing pediatric TBI clusters of high and low significance within greater Vancouver. High rate clusters tend to be concentrated to the east, while low rate clusters are most common in the west of the region. This pattern directly corresponds to Socio Economic Status (SES) values for the areas: the west side of Vancouver has consistently higher SES values as illustrated.

Pediatric Traumatic Brain Injury (TBI) data from the BC Trauma Registry (BCTR) was used to identify all pediatric TBI patients admitted to BC hospitals between the years 2000 and 2013. The primary variables within the data included patient place of residence, age, gender, injury mechanism, intent and injury severity score (ISS). All patients with an ISS of 12 and over were included in the data as such injuries are severe enough to warrant admission to hospital. All locations were geocoded using DMTI geopinpoint [14]. The study area encompassed the greater Vancouver region between West Vancouver and Hope (Figure 1). The study was conducted in accordance with the Declaration of Helsinki, and the protocol was approved by the Ethics Committees of the University of British Columbia and Simon Fraser University.

Population variables (namely, the number of children aged 0–18), at the dissemination area (DA) level were taken from the 2006 Canadian census gathered by Statistics Canada. The number of injuries within each DA was calculated using a spatial join and a crude injury rate was calculated for each DA using the population of children aged 0–18 as the denominator.

Census data at the DA level was also used to examine the relationship between SES and injury rate. Each DA is generally composed of neighbouring streets that host somewhere between 400 and 700 residents [15]. SES census variables used as potential predictors for injury rate included: percentage of the population 15 and over with no high school diploma, median income and average income (both of which were divided into 10 incremental groups beginning with 0–10,000 and continuing up to 90,000–100,000. A separate value was used for those with income over 100,000), percentage of people identified as Aboriginal within the population, unemployment rate, and the percentage of non-detached housing. All potential census variables were chosen based on the systematic review by Bell *et al.* which examined the most common SES census variables used in injury research [16]. As a separate analysis, a popular composite SES index (the VANDIX) was used to examine the relationship between pediatric TBI rate and SES. The VANDIX is composed of seven variables taken from the 2006 census and used to construct a deprivation index at the DA level. The VANDIX has been demonstrated to be a reliable predictor in the assessment of SES and health status [17]. It provides a deprivation score from 1 to 5, with 1 being low and 5 being high.

### 2.2. Geographic Variables

Two additional variables were created for use as potential predictors in both models. These included a 30 min driving time catchment around BC Children’s Hospital, the Level I Pediatric Trauma Centre and only pediatric hospital for the greater Vancouver area, and a 1000 m buffer zone around each population-weighted DA centroid. The 30-minute driving time catchment was calculated using the ArcGIS network analyst function and the DMTI Route Logistics dataset. As BC Children’s Hospital is located at the center of the city of Vancouver [18,19], all DA’s within 30 min of the hospital were considered urban and all DA’s more than 30-minutes away were considered suburban. This variable was created in order to capture disparities between east and west within Greater Vancouver (the west side is known to have higher SES) and to capture disparities between urban and suburban regions. Additionally, a buffer of 1000 m was created around each population-weighted DA, and the sum of the length of the roads within each buffer was calculated. This variable was created with the intention of providing an additional potential measure of urban *vs.* suburban as well as to provide a measure of traffic density around the population centers within each DA. 

### 2.3. Hotspot Identification

In order to examine the degree of global spatial autocorrelation within the data, the ArcGIS Global Moran I tool was used. Because the DA’s were relatively small in size, a spatial rate smoother [20], as implemented in OpenGeoDa version 1.0.1 [21], was used to adjust the crude injury rates. The spatial rate smoother uses rates from neighboring DA’s for smoothing. A Local Moran I tool was used to determine local clusters and was then implemented on the smoothed surface. 

### 2.4. Statistical Analysis

Using SPSS statistical software [22], negative binomial (NB) models were created to assess the relationship between SES and injury rate. The first, examined how both individual census variables and geographic variables correlated with injury rate. The second used the VANDIX composite SES variables together with the geographic variables to assess the same relationship. A NB model was used because the outcome variable had a Poisson distribution (many DA’s had a zero count of injuries) with over dispersion (the variance does not equal the mean) and NB models allow for this where Poisson models do not [23]. In the first model, variable selection was implemented by first examining the relationship between each individual census variable and injury rate (count/log (total kids age 0–18)). Only variables with a significant relationship at *p* < 0.2 were kept and included as potential variables in the final model. In the final model, only variables with a significant correlation of *p* < 0.05 were kept. The second model, simply examined the relationship between injury rate, as the dependent variable, and the VANDIX and geographic variables, as the independent variables. The VANDIX score was also used to explore differences in injury mechanisms between the deprived and the less deprived populations. The model residuals were examined in order to assess whether spatial dependency affected the results.

## 3. Results

### 3.1. Descriptive Analysis

Six hundred and fifty three severe pediatric TBI injuries occurred within the BC Lower Mainland between 2000 and 2013. Of the 653 injuries, 182 (27.8%) were sustained by girls. The largest gender-related difference in injury prevalence was between the age of 10 and 14 (Table 1). 335 of 653 (51.3%) TBI’s were in children with very severe injury severity scores (ISS ≥ 25) with the remaining being moderately severe. The median ISS, stratified by age, are presented in Table 1. The majority of injuries occurred in children between 15 and 18 years of age (249 of 653 (38%)). The most common causes of TBI were: motor vehicle collision (MVC) (351 of 653 (54%)), falls (170 of 653 (26%)) and assault (67 of 653 (10%)). As expected, there is a clear downward trend in injuries from falls as children get older, with falls being the primary cause of injury within the youngest age group (62%) and the least significant cause of injury within the oldest age group (7%). MVC are the number one cause of TBI’s for children in age groups 5–9, 10–14 and 15–18 (62%, 71% and 65% respectively) but are responsible for only 17% of the injuries within the youngest age group. Intentional injuries (e.g., assaults) are most prevalent in the oldest age group (23%) followed by the youngest age group (7%) (non-accidental trauma). Overall, 12% of the injuries were intentional (Table 2).

**Table 1:** Shows the distribution of Traumatic Brain Injury (TBI) by gender. Boys experience the highest rates of injury at all age ranges, but this difference is most pronounced between the age of 10 and 14.

Age Group	Girls	Boys	All	% Difference
0–4	65	97	162	19.80
5–9	27	65	92	41.30
10–14	31	119	150	58.70
15–18	59	190	249	52.60
0–18	182	471	653	44.30

**Table 2:** Shows pediatric TBI by age group, gender, injury mechanism and intent. Injuries from falls are the major cause of injuries for those aged 0–4. Intentional injuries and injuries from assault are the number one cause of injuries for those 15–18.

	Age Group (Years)				
**Median ISS (*IQR*)**	**0–4**	**5–9**	**10–14**	**15–18**	**All**
	*n = 162*	*n = 92*	*n = 151*	*n = 248*	*N = 653*
*Female*	17 (9.5)	**20 (13)**	26 (17)	26 (15)	25 (13)
*Male*	17 (9)	25 (16.5)	25 (13)	26 (16	25 (12)
**Injury Mechanism, *n* (*%*)**					
*Assault*	15 (9%)	0 (0%)	4 (3%)	48 (19%)	67 (10%)
*Fall*	**101 (62%)**	28 (30%)	23 (15%)	18 (7%)	170 (26%)
*MVC*	27 (17%)	57 (62%)	**107 (71%)**	160 (65%)	351 (54%)
*Other*	19 (12%)	7 (8%)	17 (11%)	22 (9%)	65 (10%)
**Motivation, *n* (*%*)**					
*Intentional*	12 (7%)	1 (1%)	9 (6%)	**57 (23%)**	79 (12%)
*Unintentional*	141 (87%)	91 (99%)	139 (92%)	187 (75%)	558 (85%)
*Unknown*	9 (6%)	0 (0%)	3 (2%)	4 (2%)	16 (3%)

### 3.2. Hotspot Analysis

The Global Moran I analysis indicates the data to be moderately clustered (*p* value 0.076) over the study area. After applying spatial smoothing in order to highlight global spatial trends over the study area, clustering of high and low rates of injury were clearly apparent (Figure 1). High rate clusters were identified across the eastern part of the Lower Mainland around Mission and the Fraser Valley. Additionally, high clusters were seen in areas of Coquitlam, Surrey and on the North Shore. Low rate clusters of TBI were visible on the West side of Vancouver, as well as in Richmond and Tsawwassen.

### 3.3. Statistical Analysis

#### 3.3.1. Individual Census Variables

The negative binomial (NB) regression for each individual independent variable showed a value of *p* < 0.2 for the following SES variables: average income, proportion Aboriginal, proportion with no high school diploma, DA within 30 min of BC Children’s Hospital (rural *vs*. suburban) and the sum of roads within each 1KM DA buffer. As a result, only these variables were used as potential variables in the final NB model (Table 3).

**Figure 2:**
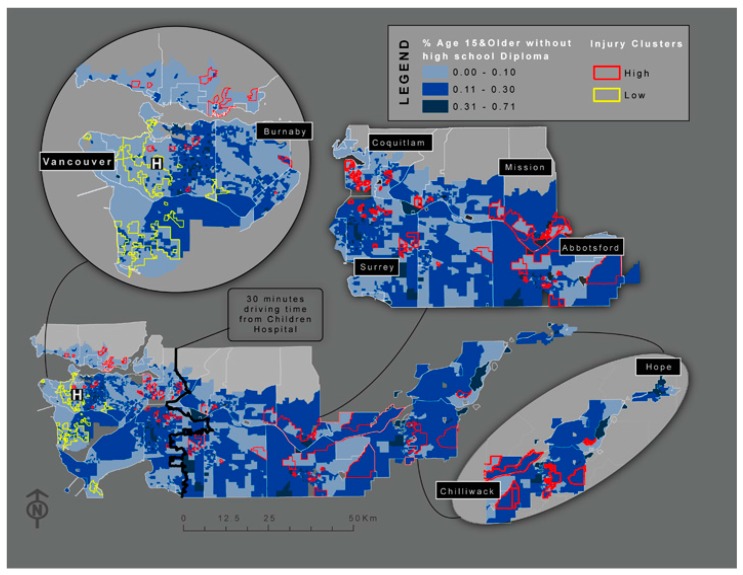
Dissemination Area (DA) level map indicating high (red) and low (yellow) clusters of TBI rates, overlapped with the percent of people age 15 and older with a high school diploma. There is a clear trend between TBI rate and possession of a high school diploma, with the highest rates of TBI occurring in areas where a very high proportion of the population did not obtain a high school diploma.

After trying several combinations of variables and comparing their AIC value, the final model included only two variables: proportion of people with no high school diploma and whether a DA was within 30-minutes of BC Children’s Hospital (Table 4). The remaining three variables were excluded as they were no longer significant (at *p* < 0.05) when combined with other variables. The final model results indicated that for each 10% increase in the proportion of people with no high school diploma the rate of injury increased by 13%. Additionally, children who lived in an area more than 30-minutes (driving time) from BC Children’s Hospital had a 21% greater chance of suffering a TBI (Table 4, Figure 2). 

**Table 3:** List of variables considered for the final model. Only SES variables with *p* ≤ 0.2 were used as potential variables in the final model.

Name	Description	OR	Group	Pass Criteria for Final Model
		(95%CI)		
		*p*-Value		
PercAboriginal	Percentage of Aboriginal	1.002	Cultural	Yes
		1.003–1.02		
		0.02		
PercLonFamily	Percentage of Lone Families	1.002	Demographic	No
		0.994–1.011		
		0.6		
PercNoHSchl	Percentage of age 15 and older with no high school certificate	1.013	Education	Yes
		1.005–1.021		
		0.001		
AllRdBfr	Sum of length of roads within 1000 m	0.999	Environmental	Yes
		0.998–1.000		
		0.003		
PercNoDetHous	Percentage of no Detached Housing	0.999	Housing	No
		0.996–1.001		
		0.35		
AvgInc	Average income	1	Income	Yes
		1.001–1.002		
		0.176		
MedInc	Median Income	1	Income	No
		1.001–1.002		
		0.41		
UnEmpRate	Unemployment rate	0.796	Occupation	No
		0.93–1.02		
		0.88		
Within30min	Within 30 min of BC Children Hospital	0.796	Rural/Suburban	Yes
		0.674–0.941		
		0.01		
VANDIX	Composite SES Index	1.089	Composite SES Index	Yes
		1.031–1.151		
		0.002		

**Table 4:** Shows the final negative binomial model for both the VANDIX and the census variables. The Exp(B) for the VANDIX model indicated that for each 10% increase in the proportion of people with no high school diploma the rate of injury increased by 13%. The Exp(B) for the census variable model indicated that with each increase in the deprivation score there was an 8% increase in the risk of TBI. Additionally, children who lived in an areas more than 30 min (driving time) from British Columbia (BC) Children’s Hospital (Children living in suburban areas) had a much greater chance of being injured in both models.

Predictor Variable	Models	
	Census Variables	VANDIX
	OR (95%CI) *p*-Value	OR (95%CI) *p*-Value
	(n = x)	(n = x)
No high school	1.13	-
	(1.03–1.23)	
	0.009	
Outside of 30 min (drive time)	1.22	1.25
	(1.01–1.48)	(1.04–1.51)
	0.039	0.020)
VANDIX	-	1.08
		(1.02–1.15)
		0.01

#### 3.3.2. Composite SES Model 

The VANDIX composite SES variable was also highly correlated with injury rate when examined both individually and together with the geographic variable (within a 30-minute drive of the hospital). The final model indicated that for every increase in the VANDIX deprivation score (from 1 to 5) the rate of injury increased by 8% (Table 3). 

An assessment of both model residuals, in terms of spatial autocorrelation, indicated that a spatial dependency did not exist (*p* > 0.05). This suggests there is no need to modify the model to address this issue.

#### 3.3.3. Injury Mechanism and SES

**Figure 3:**
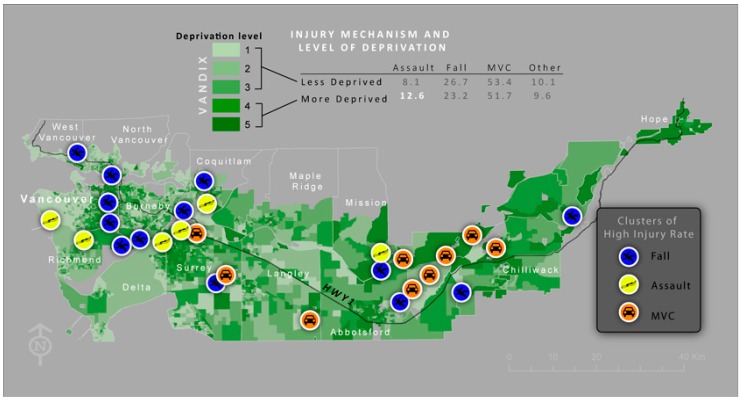
Shows clusters of high rates of TBI, by injury mechanism. TBI as a result of motor vehicle collision (MVC) tend to cluster in the eastern part of the greater Vancouver (suburban) region while assault and falls tend to occur more often in the western part.

An examination of injury mechanisms by socio-economic status, using the VANDIX deprivation score, demonstrated that 13% of the injuries in the more deprived DA’s (DA with deprivation score 4 and 5) occurred as a result of assault as opposed to 8% in the less deprived DA’s (score 1–3). Injuries from falls have an inverse relationship with SES, with almost 27% of the injuries in the higher SES areas resulting from falls *versus* 23% in the lower SES areas (Figure 3). Injuries from MVC have an almost identical percentage across the SES scores.

## 4. Discussion

This paper highlights the relationship between different SES indicators and pediatric TBI rates in greater Vancouver. A significant relationship is clearly visible when using both the VANDIX (composite SES variable) and education variables (not having a high school diploma) as predictors of injury. Income-based census variables seem to be a poor predictor of TBI rates. Even though, average income was somewhat significant (*p* = 0.176) when used individually, it had very poor predictive value when used in combination with other variables in the final model. Understanding why income is not a good predictor of injury rate is beyond the scope of this study, but it could very well be that income assistance and social programs for low income families reduce the risk of injury for these children. The results of this study can be generalized primarily to developed countries and, specifically, to locations where extreme poverty and poor living conditions are not really an issue. In such locations education level may be a better predictor for injury rate. Another variable demonstrating good prediction of injury rates is the percentage of Aboriginal people within each DA. This is not surprising, as it has been shown that children and youth residing in areas with higher percentages of Aboriginal people are twice as likely to be hospitalized when compared with children and youth in other areas. In addition, mortality rates as a result of injury are much higher amongst native children [24,25,26]. The percentage of people not having a high school diploma is likely a better predictor as it encompasses within it a large proportion of the Aboriginal population but also other vulnerable populations without education. 

Epidemiological research examining the relationship between prevalence of pediatric TBI and SES has shown similar results. Tennant examined the relationship between TBI and SES across all age groups and found hospital admission rates (including children 0–18) increased by 17.4 percent for each one percent decrease in unemployment rate (age 16–24). Similarly, admission rates increased by 11 per 100,000 for every one percent increase in single parent family rate [27]. Yates *et al.* also found a positive relationship between low SES and higher attendance in emergency departments as a result of head injury in pediatric populations. They demonstrated that children under 5 years of age in lower SES urban areas were particularly vulnerable to TBI [28]. Roberts et al, examining the likelihood of mortality from injuries, found that children in impoverished homes in England were 1.47 times more likely to die from pedestrian injury and 1.46 times more likely to die from fall injury than children with high SES, both of which are major cause of TBI’s [3]. Injury severity also shows a similar trend. A study in Ontario, Canada, found that children from low SES homes were 1.75 times more likely to be admitted to a hospital for injuries occurring within the home and 1.42 times more likely to be admitted for fall injuries. There are several reasons why this relationship exists, most notably the relatively low use of helmets, car-seats and seatbelts as safety measures amongst low SES and less educated populations [2,29,30]. This suggests that prevention strategies should be directed to these populations within the specific geographic areas where higher rates of pediatric TBI exist. 

The relationship between SES and pediatric TBI has been shown to persist even in the post-TBI rehabilitation stage. Several studies indicate that rehabilitation outcomes for children with TBI are much more favorable for children of higher SES. Yeats *et al.* indicated that the social competence scale (ability to get along with friends and siblings) of children recovering from TBIs is much lower for non-white and lower SES children. After studying the impact of TBI on children who were in injured in Ohio, they concluded that TBI rehabilitation results in poorer social outcomes for children of lower SES [13]. A similar study of TBI outcomes amongst Australian children found that low SES has an impact on long term social functioning even as children transition to adulthood [31].

The prevention of TBI’s among children and youth requires understanding of the contexts and environments in which these injuries occur. The places where children and youth live, learn, work and play have an impact on their injury experience, and those living and attending school in low SES neighbourhoods are disproportionately affected [6]. However, many of the neighbourhood and school characteristics related to risk of TBI can be modified. Efforts, such as free or subsidised safety helmet programs, and youth peer-mentorship programs that are aimed to address neighbourhood and school culture related to risk-taking attitudes and behaviours, bullying, and driver education, are examples. Continuing research programs are also needed to implement targeted injury prevention programs among low SES neighbourhood youth, including those living in suburban and more rural settings. 

## 5. Study Limitations

This study has some limitations that are important to note. First; the injuries recorded in the injury data sets are recorded by place of residence rather than place of injury. Unfortunately; place of injury is not something that is currently captured by trauma datasets. As a result; there may be some inherent errors within the driving time calculations. There is currently no way to overcome this issue. That said, previous research has noted that most injuries do occur between 5 and 10 miles of home [32,33]. In addition, as place of residence was used to extrapolate SES levels, it was necessary to use place of residence in this study. Finally, data for TBI injuries resulting in death, where the death occurs outside of the hospital, is also missing from this study. While this data is recorded in the vital statistics data for BC, it was unavailable for this study.

## 6. Conclusions

This study utilized advanced spatial analysis techniques to explore where injuries occur in high concentrations and to determine whether distinct social processes may help to explain the existence of these clusters. While there is a clear relationship between different SES indicators and pediatric TBI rates in greater Vancouver, this study indicates that income-based SES indicators do not serve as good predictors within this region. 
